# Forces applied during classical touch assays for *Caenorhabditis elegans*

**DOI:** 10.1371/journal.pone.0178080

**Published:** 2017-05-19

**Authors:** Adam L. Nekimken, Eileen A. Mazzochette, Miriam B. Goodman, Beth L. Pruitt

**Affiliations:** 1 Mechanical Engineering Department, Stanford University, Stanford, California, United States of America; 2 Electrical Engineering Department, Stanford University, Stanford, California, United States of America; 3 Molecular and Cellular Physiology Department, Stanford University, Stanford, California, United States of America; East Carolina University, UNITED STATES

## Abstract

For decades, *Caenorhabditis elegans* roundworms have been used to study the sense of touch, and this work has been facilitated by a simple behavioral assay for touch sensation. To perform this classical assay, an experimenter uses an eyebrow hair to gently touch a moving worm and observes whether or not the worm reverses direction. We used two experimental approaches to determine the manner and moment of contact between the eyebrow hair tool and freely moving animals and the forces delivered by the classical assay. Using high-speed video (2500 frames/second), we found that typical stimulus delivery events include a brief moment when the hair is contact with the worm’s body and not the agar substrate. To measure the applied forces, we measured forces generated by volunteers mimicking the classical touch assay by touching a calibrated microcantilever. The mean (61 μN) and median forces (26 μN) were more than ten times higher than the 2-μN force known to saturate the probability of evoking a reversal in adult *C. elegans*. We also considered the eyebrow hairs as an additional source of variation. The stiffness of the sampled eyebrow hairs varied between 0.07 and 0.41 N/m and was correlated with the free length of hair. Collectively, this work establishes that the classical touch assay applies enough force to saturate the probability of evoking reversals in adult *C. elegans* in spite of its variability among trials and experimenters and that increasing the free length of the hair can decrease the applied force.

## Introduction

The sense of touch is involved in almost all our daily activities, yet we do not fully understand the molecular events that enable sensory neurons to detect touch. *Caenorhabditis elegans* roundworms are used to study the sense of touch for a variety of reasons, including their well-characterized and compact nervous system, vast genetic toolkit, and deterministic cell lineage [1]. The classical touch assay is the most common method for measuring the touch sensitivity of wild-type and mutant worms. In this assay, an experimenter touches the animal with a fine eyebrow hair and observes whether or not the animal changes direction of movement by performing a reversal [2–11]. A worm is considered insensitive to gentle touch if it fails to respond to stimulation with an eyebrow hair, but responds to more vigorous stimulation with a platinum wire (harsh touch) [5]. One significant limitation of the classical gentle touch assay is that the mechanical stimuli delivered during this assay is neither controlled nor quantified. In this study, we determine the forces applied in the classical touch assay qualitatively using high-speed video and quantitatively by measuring the forces applied to a force-sensing cantilever.

**Fig 1:**
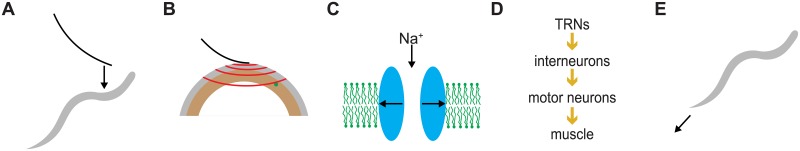
The *C. elegans* touch response encompasses the entire mechanotransduction pathway from mechanical stimulus to behavior. (A) In the classical touch assay, a stimulus is applied with an eyebrow hair, (B) force is transmitted through the worm, (C) ion channels regulate depolarization of a touch neuron, (D) interneurons pass the signal between the touch neuron and motor neurons, and (E) motor neurons activate body wall muscles to produce a behavioral response.

The input for the classical touch assay is a force on the worm’s cuticle and the output is whether or not the worm performs a reversal. Many factors can influence the outcome of an individual trial, as shown schematically in Fig 1. First, an experimenter applies a stimulus with an eyebrow hair glued to a toothpick. Next, force is transmitted through the skin to touch receptor neurons (TRNs) to activate mechanosensitive ion channels and thus depolarize the TRNs. Finally, this electrical information is transferred to interneurons and motor neurons that activate body wall muscles to produce a reversal. Among other applications, the classical touch assay has been used successfully in concert with other methods to map the touch circuitry of *C. elegans* [4].

**Fig 2:**
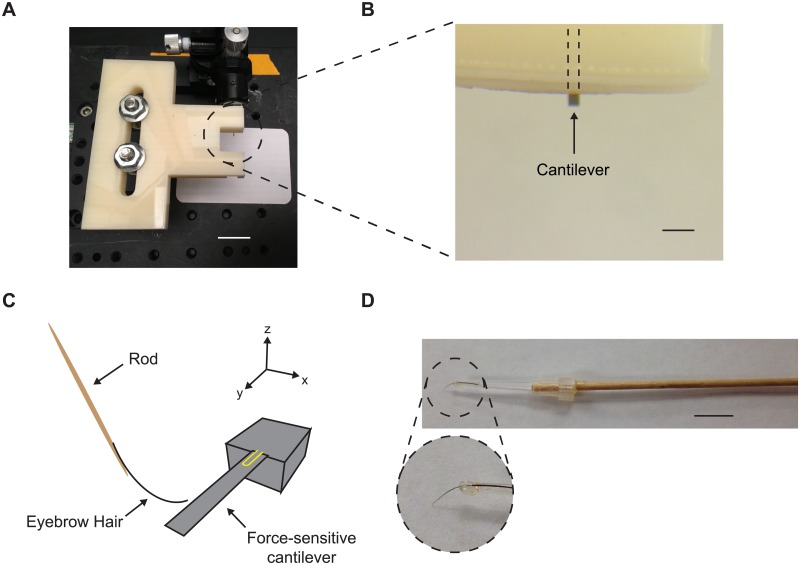
Set-up for emulating the classical touch assay and measuring mechanical forces applied by an eyebrow hair tool. (A) The cantilever is behind a piece of acrylic that limits force application to the cantilever tip and protects the cantilever from damage. Scale bar 30 mm. (B) Close-up of the cantilever behind protective acrylic. Scale bar 100 μm. (C) Volunteers touched the cantilever with an eyebrow hair, mimicking the classical touch assay. (D) All volunteers used the same hair apparatus to eliminate hair mechanics as a variable in this experiment. Scale bar 10 mm.

Previously, we deployed force-sensitive microcantilevers in a feedback-controlled system for applying defined forces to worms and used this system to study body mechanics [12–14] and behavioral responses [14]. The microcantilevers are fabricated from silicon and contain a doped piezoresistor that changes its electrical resistance as a function of mechanical stress. Here, we used microcantilevers to make quantitative force measurements to provide a basis for comparison between studies using microcantilevers and those relying on the classical touch assay. To achieve this goal, we measured the forces applied by human volunteers emulating the classical assay (see Fig 2) and quantified variations in the stiffness of eyebrow hairs derived from several human donors. High-speed videos indicate that there is an instant where the entirety of the mechanical load is applied to the top of the worm, so we used the vertical force applied by the hair to the cantilever as a measurement of the force applied to the worm. Our results indicate that both expert and novice human volunteers deliver forces that are consistently higher than the 2-μN force known to saturate the probability of a touch response in *C. elegans* [14]. Collectively, these results reinforce the idea that this assay has the power to detect touch impairment, but not sensitization.

## Results

### High-speed videos demonstrate how force is applied to the worm

**Fig 3:**
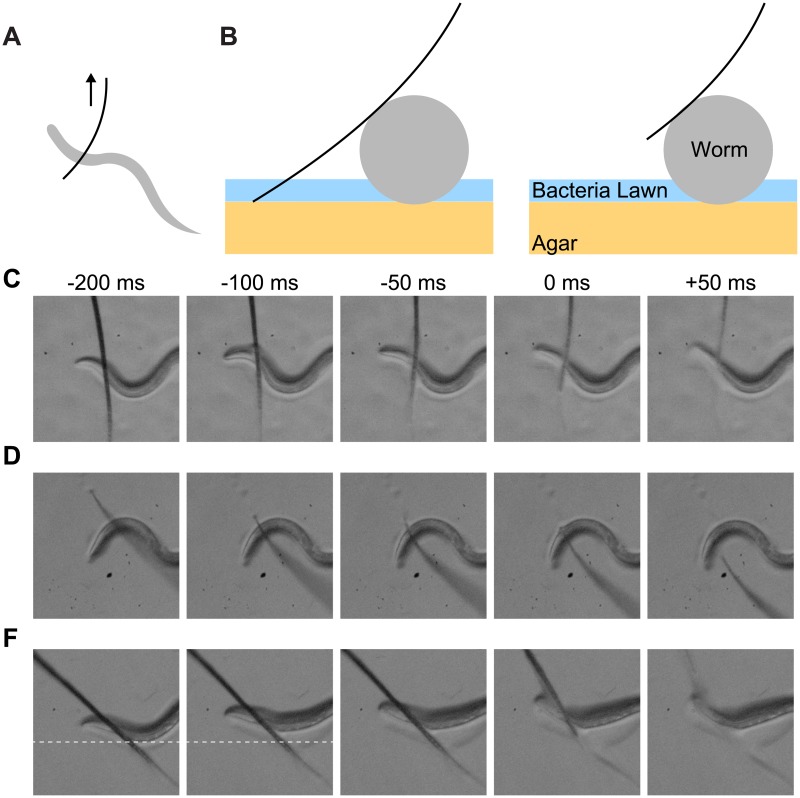
Representative examples of high-speed videos that indicate maximum force is applied to the worm. (A) Schematic representing the hair moving across the worm. (B) Cross-section showing when the hair first comes into contact with the worm. At this instant, it is also in contact with the agar and bacterial lawn. (C) As the hair moves across the worm, it lifts off the agar and is only in contact with the worm. (D-E) Images from representative videos of the hair sliding over the worm, temporarily lifting off the agar. (F) In this case, the worm is pushed across the agar laterally before the hair slides over the top of the worm. The dashed line reference shows lateral movement. Times are in reference to the time when the hair is only in contact with the worm as in panel C.

We recorded 10 high-speed videos of the touch assay being performed and used the video stacks to investigate the nature of the interaction between the hair and a freely moving worm crawling in a bacterial lawn on an agar substrate In all of the videos, the hair first touches the agar and then slides to contact the worm. In 7 of the videos, the hair proceeds to slide over the top of the worm (Fig 3 and S1 Video). In the other 3 videos, the hair pushes the worm sideways through the bacteria lawn before sliding over the top of the worm. Experts would recognize the former sequence as typical of the assay and the latter sequence as an error in assay performance. In both of cases, however, the hair momentarily loses contact with the agar entirely before touching down again on the other side of the worm. During this moment, all of the force proportional to elastic bending of the hair is applied to the top of the worm.

### Eyebrow touch forces exceed forces sensed by worms

**Fig 4:**
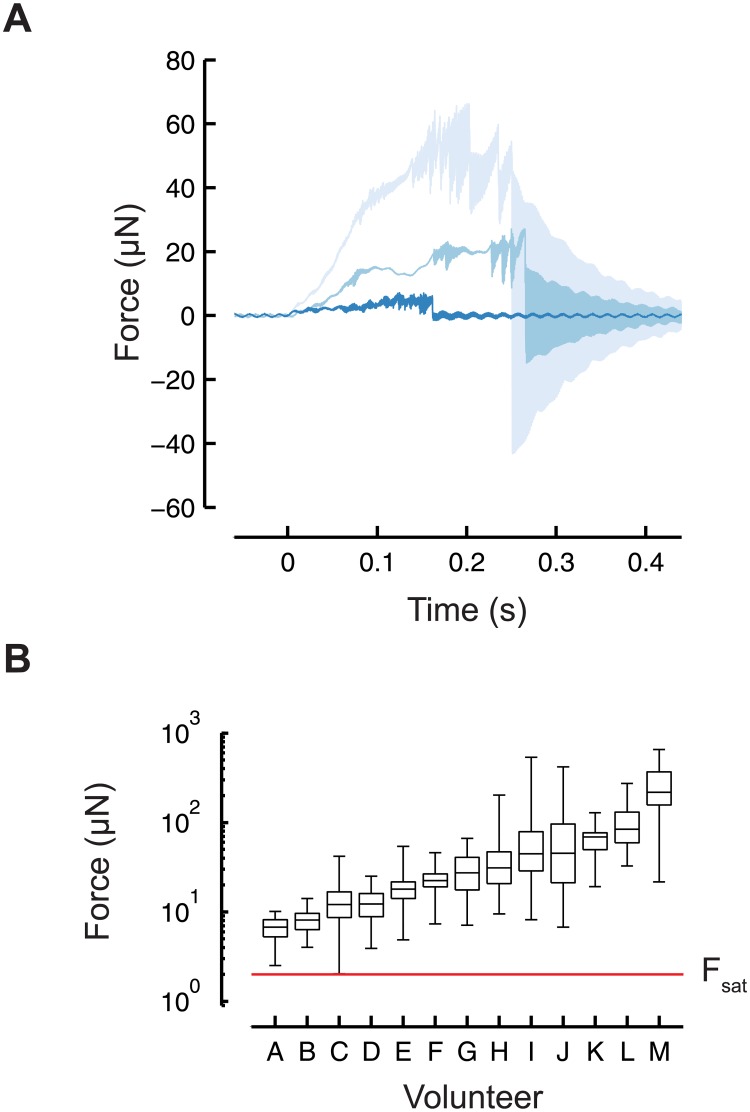
Forces applied by volunteers all exceeded the force required to produce the maximum probability of response from a worm. (A) Volunteers used an eyebrow hair to touch a force-sensing cantilever 30 times. A sensing circuit records the z-force applied to the cantilever as a function of time. The traces plotted here are the minimum, median, and maximum for touch events for the median volunteer (volunteer G). (B) Box plots of forces delivered by human volunteers. The central mark is the median, the edges of the boxes are 25th and 75th percentiles, and the whiskers indicate the minimum and maximum forces. The red line indicates the force at which the probability of a worm reversing is saturated (about 2 μN, F_sat_).

Using a force-sensing microcantilever similar to ones reported previously [15], we measured the forces applied by volunteers (Fig 2A and 2B, Methods). We were able to detect touch events that delivered forces greater than 0.5 μN and less than 1.1 mN (see Methods for details). Each volunteer performed 30 trials using the same eyebrow hair (see S1 Fig). Fig 4 shows the forces applied by the volunteers. For comparison (Fig 4B, red line), a 2-μN force is sufficient to saturate the behavioral response of wild-type worms [14]. Thus, the forces applied by all human volunteers were consistently higher than those needed to reliably evoke behavioral responses.

Next, we examined forces delivered as a function of trial number. We did not detect any general trends in forces over the course of the experiment. Pearson’s correlation coefficient was positive for five volunteers and negative for the other eight volunteers, indicating that some individuals tended to apply more or less force as the experiment progressed, but this trend was not consistent between volunteers.

The forces were log-normally distributed for all volunteers (Lilliefors test on ln(Force), 5% significance level), and the median force from all trials was 26 μN. The coefficient of variation of the distribution was 1.7, and the mean force was 61 μN.

### Eyebrow hairs as a source of variability in classical touch assays

**Fig 5:**
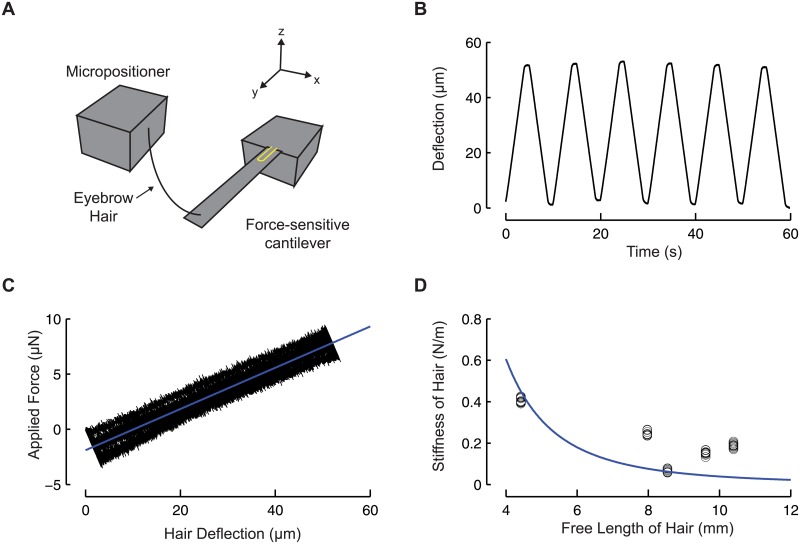
The mechanical properties of eyebrow hair tools. (A) Schematic of the method for measuring the stiffness of an eyebrow hair. (B) Representative trace of the deflection of the hair as a function of time. (C) Representative force-displacement curve. At least four curves were collected for each eyebrow hair. The slope of this curve is the stiffness. (D) Stiffness vs. length of the hair. Points are the measurements from five different hairs, and the smooth line was fit to the data according to Eq 1.

The force applied to worms during the classical touch assay depends not only on the experimenter, but also on the stiffness of the hair itself. We used force-sensing microcantilevers to measure the stiffness of single eyebrow hairs from five donors to examine this source of variance (Fig 5). First, we attached an eyebrow hair to a micropositioner stage and moved it close to the cantilever without coming into contact (Fig 5A). Next, we bend the hair against the cantilever by moving the micropositioner (Fig 5B). The hair’s stiffness is the force applied to it divided by its deflection (slope of Fig 5C). Stiffness values varied between 0.067 N/m and 0.41 N/m (Fig 5D).

Four of the hairs were glued such that the free length of the hair beyond the glue point was maximized. This configuration will minimize stiffness, k (N/m), which relates force F (N) and displacement d (m) by Hooke’s law, *F* = *kd*. To a first approximation, we assume the hair behaves as an isotropic, linear elastic cantilevered beam of constant diameter D, where E is Young’s modulus, and L is the length of the beam (Eq 1).

$$\begin{array}{c} k=\frac{3E\pi D^{4}}{64L^{3}} \end{array}$$ (1)

The stiffest hair was glued according to the traditional method described in [10]. The stiffness of the hairs does decrease with increasing length (Fig 5D). The discrepancy between the data and the theoretical fit (Eq 1) suggests that differences in cross-section, material properties, and deviations from isotropy and linear elasticity also contribute to variations in stiffness.

## Discussion

An ideal assay for touch sensation would consistently identify touch defects with high throughput and low cost. The classical touch assay for *C. elegans* nematodes can be performed without any specialized equipment other than a microscope and requires minimal training and experience to perform. These features make it relatively high throughput and low cost.

However, this assay cannot detect minor defects in touch sensitivity. The forces applied by volunteers to a cantilever in our experiments exceeded the force that saturates the probability of a behavioral response in the worm [14]. This force may be further increased during a touch assay by the presence of surface tension, which can pull the hair towards the worm or agar plate. It is possible that the maximum force occurs before or after the hair is only in contact with the worm. In this case, our measurements of the force applied to the cantilever would overestimate the force applied to the worm. The subset of high-speed videos that showed the worm being pushed laterally by the hair indicates that the lateral force may be significant in some cases.

Other drawbacks include variability in forces delivered due to differences in the mechanical properties of the eyebrow hair tool or in the performance of different experimenters. A longer hair with a smaller cross-sectional area will have a lower overall stiffness. Differences in hair material properties and geometry also contribute to the stiffness, but stiffness maintains the same proportionality to length in this case (*k* ∝ *L*^−3^). Notably, the tool we provided to thirteen volunteers was softer than the others tested. If the stiffness of the eyebrow sets the range of forces applied by volunteers, then force would be increased by a factor of six if the human volunteers had used the stiffest hair in our test set.

Despite these drawbacks, the classical assay for *C. elegans* touch sensation will continue to inform our understanding of the genetic basis of touch sensation. Our results indicate that the classical touch assay saturates the probability of a reversal in every trial across all experimenters, making it easy to differentiate severe touch defects, even when different people perform the assay. This assay should be used in concert with other methods such as electrophysiology or calcium imaging to study subtle touch defects, however. Methods for measuring stimulus-response curves for worms and other small, soft animals (e.g. [14]) could also be deployed in this case.

## Conclusion

The classical touch assay is a simple, qualitative way to differentiate the touch sensitivity of nematodes. The sensitivity of the assay is limited by the delivery of forces greater than the force required to maximize the worm’s probability of response, variation in force applied by different experimenters performing the assay and across trials delivered by a single individual, and by variations in the mechanics of eyebrow hairs. If the hair is glued such that its free length is increased, then the experimenter will apply smaller forces to the worm. This maneuver could increase the sensitivity of the assay for detecting minor touch defects. These considerations should be taken into account when designing experiments using the classical eyebrow hair touch assay to study touch sensation and comparing results gathered by different researchers.

## Materials and methods

### High-speed video

We recorded high-speed video of several touch assays using a Phantom VEO 640S camera (Vision Research) on a stereomicroscope equipped with a trinocular camera port. The videos were recorded at 2500 frames per second with an exposure time of 390 μs. The images were originally 2048 × 1152 pixels, then they were later cropped using the same size window in the same location within the each image. We rescaled the range of all pixel values from the original 0–255 to 0–127 to improve contrast.

### Forces applied by volunteers

Volunteers were shown a video of a touch test (youtube.com/watch?v=olrkWpCqVCE), then given a plate with 3–5 free-moving worms to practice the touch test. Next, they were presented with the end of a force-sensitive microcantilever under a stereomicroscope (Fig 2B). The cantilever was oriented to be most sensitive to vertical forces (z-axis). Volunteers were instructed to touch the cantilever with a vertical motion using the fine hair attached to a slender wooden rod (Fig 2D). We told the volunteers to touch the cantilever gently, emulating the gentle touch assay as much as possible. We constructed and built the eyebrow hair tool. The same apparatus and eyebrow hair tool were used by all human experimenters.

Forces applied during hair touch assays were measured using previously reported micromachined transducers [16]. These sensors were silicon cantilevers with a doped piezoresistor that changes resistance proportional to strain. The change in resistance was measured using a Wheatstone bridge, where one half of the bridge contained a potentiometer for balancing the bridge. Two of the other sides of the bridge contained fixed resistors and the final side had the sensing cantilever. The bridge’s output was amplified using an instrumentation amplifier (INA103) with a gain of 500 or 1000. For each volunteer, we initially set the gain to 1000, and then decreased it to 500 if the forces applied by the volunteer were close to the maximum force that could be detected. The maximum force the system could measure was 1.1 mN when the gain was 500 and 550 μN when the gain was 1000. The minimum force the system could measure was 0.9 μN when the gain was 500 and between 0.5 and 1 μN when the gain was 1000. This minimum force detection threshold is set empirically during data analysis based on the noise of a given trial, as described below. A passive (RC) low-pass filter with a cutoff frequency of 2.3 kHz was used to reject high frequency noise.

We collected data with a LabJack U6-Pro data acquisition module at a sampling rate of 5 kHz using custom software written in Python. After the data were collected, we detected touch events by computationally filtering the cantilever signal with notch filters at 60 Hz and 1400 Hz (30th order digital Butterworth filters) to remove line noise and ringing from the cantilever, respectively, and a 5 Hz high pass filter (Equiripple Highpass filter generated using the FIRPM function) to remove drift. We designed all three filters using MATLAB’s Filter Design and Analysis Tool. We then classified any signal exceeding three times the standard deviation of the remaining noise as a touch event. After detection of a touch event, we manually confirmed that the touch event was valid. There were four criteria that could lead to rejection of a touch event: (1) The peak was in the wrong direction due to a force accidentally being applied from below the cantilever, (2) the detected peak was a filtering artifact such as passband ripple, (3) the touch event was too soon after a prior event to measure the baseline for drift correction, or (4) the touch event was not contained within the bounds of the detection interval. If the fourth criterion for rejection was met, we manually adjusted the detection interval. We initially set the detection interval to 1 second and increased it up to 1.5 or 2.5 seconds if a given volunteer’s touch events did not fit within a 1 second window. After detection of touch events, we used the unfiltered signal to determine the applied forces. We corrected these data for nonzero bridge balancing and drift by subtracting the mean value of the 500 measurements just before the hair touched the cantilever. The forces we report here are the maximum downward forces applied during each touch event.

### Hair stiffness measurement

We attached an eyebrow hair tool to a micropositioner stage (Physik Instrumente, Model 622.ZCL) and moved it close to the cantilever without coming into contact (Fig 5A). We then moved the position of the stage up to 60 μm over 4 seconds, held it in place for 1 second, and then ramped back to its original position over 4 seconds (Fig 5B). This displacement profile was repeated at least 4 times for each hair, giving a total of at least 8 ramps. The hair was positioned such that it did not slip when in contact with the cantilever. Trials where the hair slipped were excluded.

We used a cantilever of the same design as the other experiments, with similar signal conditioning circuitry. We used our existing force clamp system [15] to acquire data since it already features integrated control of the micropositioner stage.

### Human subjects approval

The Stanford Institutional Review Board approved this research (protocol number: 29299). We obtained written consent from volunteers prior to their participation in our study.

## Supporting information

S1 Fig All touch events for volunteer G. Touch events are sorted from largest force (top left) to smallest force (bottom right). Upon unloading of most touches, the cantilever rings. The stimuli delivered by subjects was generally between 80 ms and 1 s in duration and unimodal (one peak) with a slower loading rate and quick unloading. There were some touch events that lasted longer, up to 2.2 s or had more than one peak (not pictured here).
(EPS)

S1 Video Representative video of touch assay. This is the full video from which the images in panel A of Fig 3.
(AVI)

S1 Data Peak forces for all touches from volunteers. CSV file containing the maximum force for all 30 touches from each volunteer.
(CSV)

S2 Data Raw force data. These text files contain raw voltage data collected as described in the Methods section. The analysis scripts in S1 Analysis Code were used to process these data.
(ZIP)

S1 Analysis Code Code used to analyze force data. The matlab code used to do all data analysis is contained in this zip file. Also requires S2 Data.
(ZIP)
